# RAF1 in AgRP neurons involved in the regulation of energy metabolism *via* the MAPK signaling pathway

**DOI:** 10.7555/JBR.39.20250114

**Published:** 2025-05-28

**Authors:** Yuqian Chen, Lianci Ren, Xinyi Xu, Zhenning Sun, Mingxi Dai, Yin Li, Xiang Ma, Juxue Li

**Affiliations:** 1 State Key Laboratory of Reproductive Medicine and Offspring Health, Nanjing Medical University, Nanjing, Jiangsu 211166, China; 2 Jiangsu Provincial Key Laboratory of Molecular Targets and Intervention of Metabolic Disease Nanjing Medical University, Nanjing, Jiangsu 211166, China; 3 Clinical Center for Reproductive Medicine, the First Affiliated Hospital of Nanjing Medical University, Nanjing, Jiangsu 210029, China; 4 The Affiliated Eye Hospital, Nanjing Medical University, Nanjing, Jiangsu 210029, China; 5 The Second Affiliated Hospital of Nanjing Medical University, Nanjing, Jiangsu 210011, China

**Keywords:** RAF1, AgRP neurons, MAPK signaling pathway, CREB, obesity

## Abstract

V-raf-leukemia viral oncogene 1 (RAF1), a serine/threonine protein kinase, is well established to play a crucial role in tumorigenesis and cell development. However, the specific role of hypothalamic RAF1 in regulating energy metabolism remains unknown. In this study, we found that the expression of RAF1 was significantly increased in hypothalamic AgRP neurons of diet-induced obesity (DIO) mice. Under normal chow diet feeding, overexpression of *Raf1* in AgRP neurons led to obesity in mice characterized by increased body weight, fat mass, and impaired glucose tolerance. Conversely, *Raf1* knockout in AgRP neurons protected against diet-induced obesity, reducing fat mass and improving glucose tolerance. Mechanistically, *Raf1* activated the MAPK signaling pathway, culminating in the phosphorylation of cAMP response element-binding protein (CREB), which enhanced transcription of *Agrp* and *Npy*. Insulin stimulation further potentiated the RAF1-MEK1/2-ERK1/2-CREB axis, highlighting RAF1's role in integrating hormonal and nutritional signals to regulate energy balance. Collectively, these findings underscore the important role of RAF1 in AgRP neurons in maintaining energy homeostasis and obesity pathogenesis, positioning it and its downstream pathways as potential therapeutic targets for innovative strategies to combat obesity and related metabolic diseases.

## Introduction

The rising global prevalence of obesity poses a major public health challenge as it is a key risk factor for multiple chronic diseases such as type 2 diabetes, cardiovascular diseases, hypertension, and certain cancers^[1–2]^. As a systemic metabolic disorder, obesity is closely related to the central endocrine system, which plays a pivotal role in regulating energy balance, appetite, and metabolism^[3]^. The hypothalamus, a critical brain region, acts as the control center for energy homeostasis by integrating hormonal and nutritional signals from peripheral organs such as the pancreas, adipose tissue, and gastrointestinal tract, thereby regulating food intake, energy expenditure, and overall metabolic balance^[4]^. The hypothalamus receives input from peripheral signals, including hormones like leptin and insulin that convey the body's nutritional and energetic status, modulating central pathways in the brain to regulate food intake and energy expenditure, ensuring that energy homeostasis is maintained^[5–7]^. In the arcuate nucleus (ARC), located on both sides of the third ventricle in the hypothalamus, two distinct neuronal populations play critical roles in regulating energy balance: pro-opiomelanocortin (POMC) neurons and agouti-related peptide (AgRP) neurons^[8]^. POMC neurons are mainly involved in suppressing appetite and promoting energy expenditure through the release of anorexigenic peptides like alpha-melanocyte-stimulating hormone (α-MSH), which act on melanocortin receptors^[9]^. In contrast, AgRP neurons stimulate appetite and reduce energy expenditure by releasing orexigenic peptides such as AgRP and neuropeptide Y (NPY)^[10–11]^. This finely tuned balance between POMC and AgRP neurons underscores the critical role of the ARC in central energy regulation. Disruptions in the signaling pathways of these neurons, whether due to genetic, environmental, or metabolic factors, can lead to dysregulation of energy homeostasis, contributing to the development of obesity and related metabolic disorders^[12–13]^. While the roles of key hormones such as leptin, insulin, and ghrelin in regulating POMC and AgRP neurons are well understood, the precise intracellular signaling cascades they activate, and how these signals are integrated within complex neuronal networks, are not fully understood.

RAF1 is a serine/threonine protein kinase that shares structural and functional homology with the human *RAF1* proto-oncogene. It plays a pivotal role in cellular signaling pathways and is highly expressed in various tissues, including the brain, liver, and adipose tissue^[14]^. RAF1 was initially identified as an important molecular link between guanosine triphosphatase (GTPase) and mitogen-activated protein kinase (MAPK) modules, positioning it as a key player in intracellular signaling pathways. As a key component of the MAPK signaling pathway, RAF1 orchestrates diverse cellular processes, including cell proliferation, cell differentiation, apoptosis, and carcinogenic transformation^[15–16]^. Despite the roles established in cellular processes, mounting evidence has shown that RAF1 plays a critical role in metabolic regulation^[17–18]^. The RAF1/ERK signaling pathway, in particular, has been shown to enhance insulin secretion from pancreatic β-cells, suggesting its involvement in maintaining glucose homeostasis^[19–21]^. In the hypothalamus, the MAPK pathway is activated by various metabolic and hormonal cues, including leptin, insulin, and ghrelin^[20,22–24]^, which modulate the activity of key neuronal populations, such as AgRP and POMC neurons. Studies have shown that dysregulation of the MAPK pathway in the hypothalamus can lead to impaired insulin signaling, increased food intake, and altered energy expenditure, exacerbating metabolic imbalances^[23]^. It remains unclear whether RAF1 interacts with hypothalamic POMC and AgRP neurons through the MAPK signaling pathway and how this interaction influences the molecular mechanisms underlying metabolic regulation.

This study aimed to investigate the role of RAF1 in hypothalamic AgRP neurons regarding energy homeostasis regulation. To do so, we first examined the expression of *Raf1* in AgRP and POMC neurons of diet-induced obesity (DIO) mice. Next, we used genetic mouse models to manipulate *Raf1* expression in AgRP neurons, including overexpressing and knocking out *Raf1*. We also intended to explore the underlying mechanism by studying the connection between *Raf1* and the MAPK-CREB pathway, along with the pathway's relation to AgRP transcription and appetite-related processes.

## Materials and methods

### Animals

Eight-week-old male C57BL/6J mice were obtained from the Animal Core Facility of Nanjing Medical University, while *Agrp*-*IRES*-*Cre* (JAX stock #012899) and *Npy*-*hrGFP* (JAX stock #006417) mice were obtained from the Jackson Laboratory, USA. All animals were housed in an SPF-level barrier facility at the Animal Core Facility of Nanjing Medical University, maintained at 20–24 ℃ with a 12-h light/dark cycle, and provided *ad libitum* access to food and water. Normal chow diet (NCD, 9.4% kcal from fat) and high-fat diet (HFD, 60% kcal from fat) were obtained from Xietong Bioscience (Nanjing, Jiangsu, China) and Research Diets (New Brunswick, NJ, USA), respectively. The HFD feeding protocol for the high-fat mouse model began three weeks after adeno-associated virus (AAV) injection. Unless otherwise specified, male mice were used exclusively in this study.

### gRNA screening and AAV production

For the CRISPR-Cas9-mediated gene editing, the target regions within the genome were the first and third exons of the *Raf1* gene in mice with a C57BL/6 background. The single guide RNAs (sgRNAs) were designed using the CRISPOR website (http://crispor.tefor.net/) to minimize off-target effects. The sgRNAs used in this study were as follows: sgRNA1 (5′-TTTGCAGGTCAATGTGCGGAA-3′), sgRNA2 (5′-CTGATTGGAGAAGAACTGCAA-3′), sgRNA3 (5′-TGAAGGTGAGAGGCCTGCAGC-3′), sgRNA4 (5′-GAAGCTGAAACTTACAAAGTT-3′), sgRNA5 (5′-AAACTTACAAAGTTGTGAGTT-3′), sgRNA6 (5′-TCTGTCAGAAGTTCCTGCTAA-3′), and sgRNA7(5*'*-GTACCTACTATGTGTGTGGAC-3*'*).

These sgRNAs were individually cloned into the lentiviral transfer vector pCK002_U6-Sa-sgRNA(mod)_EFS-SaCas9-2A-Puro_WPRE (plasmid #85452, Addgene, Watertown, MA, USA). Lentiviral particles were generated by co-transfecting 293T cells with core vectors and packaging vectors (pMDL [plasmid #12251, Addgene], pVSVG [plasmid #8454, Addgene], and pRSV-Rev [plasmid #12253, Addgene]). The transfection was performed using polyethylenimine reagent (Polysciences, Warrington, PA, USA), and the medium was changed six hours later. Virus supernatant was harvested at 24 h and 48 h post-transfection and then used to infect mHypoE-N42 (N42) cells, followed by selection in DMEM containing puromycin (2 µg/mL) for five days. The genomic sequences flanking the sgRNA target sites were amplified by PCR with Q5 High-Fidelity DNA Polymerase (NEB, Ipswich, MA, USA). The PCR fragments were purified and incubated with T7 endonuclease Ⅰ (NEB) at 37 ℃ for 15 min, followed by gel electrophoresis on a 2% agarose gel. Editing efficiency was calculated using the following formula: Editing efficiency = 100% × [1 − (1 − % of cleavage product)^1/2^]. The sgRNA1 and sgRNA2 with the highest editing efficiency were selected for further study.

The cDNA of the *Raf1* gene was cloned into the multiple cloning site of the overexpression AAV plasmid backbone: pAAV-EF1a-DIO-mCherry (plasmid #50462, Addgene). In this construct, the mCherry coding sequence was replaced with the *Raf1* cDNA. The sgRNAs were respectively cloned into the knockout AAV plasmid backbones: pX601-AAV-CMV::NLS-SaCas9-NLS-3×HA-bGHpA;U6::BsaI-sgRNA (plasmid #61591, Addgene) and pAAV-FLEX-SaCas9-U6-sgRNA (plasmid #124844, Addgene). Recombinant AAV9 viral particles were generated by co-transfecting AAV-293T cells with sgRNA plasmids and virus packaging vectors pAAV2/9n (plasmid #112865, Addgene) and pAdDeltaF6 (plasmid #112867, Addgene). Cell transfection was performed using polyethylenimine reagent. The cell culture medium was changed 10 h after transfection, and the transfected cells and cell culture supernatant were harvested after an additional 72-hour culture. AAVs were purified and concentrated by iodixanol density-gradient ultracentrifugation. The concentrated AAVs were diluted with PBS to 1 × 10^13^ genome copies/mL for *in vivo* experiments. Viral aliquots were stored at −80 ℃ before stereotaxic injection.

### Macroscopic dissection of the mouse hypothalamus

After the mouse was euthanized in accordance with institutional guidelines, a midline cervical incision was made to expose the skull. Fine scissors were used to carefully remove the calvarium, and the brain was harvested. Key anatomical landmarks were identified: the optic chiasm (rostral boundary) and mammillary bodies (caudal boundary). The pituitary gland (if present) was gently detached to isolate the hypothalamic base.

Two coronal cuts were made with a scalpel: the first 1 mm caudal to the optic chiasm and the second just rostral to the mammillary bodies. This defined the rostro-caudal extent of the hypothalamus. The cerebral hemispheres were separated along the midline to expose the third ventricle. Dorsal tissues (*e.g.*, cortex and thalamus) were then trimmed away while keeping the dissection plane ventral to the fornix. Forceps were used to lift the ventral brain tissue between the two coronal cuts, ensuring the block included the arcuate nucleus region and basal hypothalamus.

The dissected tissue was immediately transferred to a pre-chilled microcentrifuge tube, flash-frozen in liquid nitrogen, and stored at −80 ℃ for subsequent analyses such as RNA extraction or immunohistochemistry.

### Stereotaxic surgery and drug administration

Eight-week-old male mice were anesthetized in a gas induction chamber using 2.5%–3% isoflurane (Cat. #R510-22-10, RWD, Shenzhen, China) in oxygen (500 nL per side) and then secured on a stereotaxic apparatus (RWD). For hypothalamic ARC targeting, the bregma was used as the reference point, and coordinates were determined based on the mouse brain atlas (AP = −1.5 mm, ML = ±0.3 mm, DV = −5.85 mm). A microinjector (RWD) was used to bilaterally inject the virus into the ARC. Following the injection, mice were allowed to recover for two weeks before subsequent experiments.

For third ventricle cannulation, a 3.8 mm cannula (Cat. #62004, RWD) was implanted stereotaxically at coordinates 2.0 mm posterior to the bregma and 5.0 mm below the skull surface under similar anesthetic conditions. Following surgical recovery, the mice were housed individually for one week. After this recovery period, they were subjected to a 12-h fasting protocol. Subsequently, insulin (2 mU, MedChemExpress, New Jersey, USA) was administered directly into the third ventricle of each mouse, and *in vivo* analysis of signaling pathways was performed 30 minutes post-injection.

### Measurement of the metabolic parameters

For virus-transduced experiments, animals were allowed to recover for three weeks after surgery to enable sufficient expression of the AAV-expressing transgene. Body weight and food intake were measured weekly.

Six weeks after virus injection, mice underwent metabolic phenotyping in a PhenoMaster system (TSE Systems, Homburg, Germany) (commissioned by the Animal Core Facility of Nanjing Medical University). Metabolic indicators measured included energy expenditure, respiratory exchange ratio, drink, feed, weight, O_2_, CO_2_, and temperature.

When the long-term weight measurement was completed, each mouse was placed in a Bruker Minispec mq7.5 Benchtop NMR Analyzer (provided by Nanjing University of Science and Technology) to measure body composition (fat mass, lean mass, and fluid, *etc.*), and the data were recorded for statistical analysis.

### Glucose tolerance test (GTT)

After a 12-hour fast, mice received an intraperitoneal (i.p.) injection of 1.5 g/kg (animals on NCD) or 1.2 g/kg (animals on HFD) dextrose in 0.9% NaCl. Blood glucose was measured before and at 15, 30, 45, 60, 90, and 120 min after injections. Blood glucose values were determined in a drop of blood sampled from the tail using an automatic glucose monitor (Accu-Check; Roche Applied Science).

### Measurement of serum leptin and insulin

The mice were fasted for 12 h following the long-term body weight measurement. One hundred microliters of tail blood was collected from each mouse at 4 ℃ and left to stand for 4–6 h. Subsequently, the blood was centrifuged at 1000 *g* for 15 min at 4 ℃ to obtain the serum. Serum insulin and leptin levels were determined using an ultra-sensitive mouse insulin ELISA kit (Cat. #MS100, Ezassay, Shenzhen, China) and a mouse leptin ELISA kit (Cat. #90030, Crystal Chem, IL, USA), respectively.

### Established genetically modified cell lines and treatment

*Raf1* cDNA fused to a FLAG tag (Cat. #MA1-91878, Invitrogen, Carlsbad, CA, USA) was cloned into the lentivirus plasmid pLVX-puro_GCaMP6s (plasmid #164589, Addgene) to generate lentivirus for infecting N42 cells and obtaining *Raf1*-overexpressing cell lines. The sgRNAs with the highest editing efficiency were cloned into the lentivirus plasmid pCK002_U6-Sa-sgRNA(mod)_EFS-SaCas9-2A-Puro_WPRE to generate lentivirus for infecting N42 cells and obtaining *Raf1*-knockout cell lines. *Raf1*-overexpressing and *Raf1*-knockout N42 cells were cultured and grown to a confluent state. When the cell density reached 80%, the cells were starved for 12 h in serum-free medium. Insulin (5 nmol/L, MedChemExpress) was then added to the cells and incubated for 30 min. After being washed with phosphate-buffered saline (PBS) twice, the cells were lysed using RIPA lysis buffer (containing protease and phosphatase inhibitors). The total and phosphorylated proteins were then detected.

### Fluorescence *in situ* hybridization (FISH) assay

The brain tissue was fixed with 4% paraformaldehyde (PFA), embedded in optimal cutting temperature (OCT) embedding medium on liquid nitrogen, and stored at −80 ℃ until cryostat sectioning (16 μm thickness) onto Adhesion Microscope Slides (Citotest, China) for detection of *Raf1* and *Agrp* mRNA expression. The targeted region for the *Raf1* probe was 697–876, accession #NM_001356333.2; the *Agrp* probe targeted region 321–717, accession #NM_001271806.

For the staining process, sections were treated with preheated proteinase K (10 μg/mL) for 5 min at 37 ℃, followed by inactivation of proteinase K with 4% PFA. Then, the probe solution was dispensed onto the slide and incubated at 37 ℃ overnight. On the following day, the fluorescence amplification reaction solution was applied to the slide and incubated at room temperature for 8 h. Thereafter, the fluorescence amplification reaction solution was rinsed off, and the slide was affixed using ProLong Glass Antifade Mountant with NucBlue (Invitrogen). Finally, an LSM800 confocal microscope (Carl Zeiss, Jena, Germany) was utilized to capture fluorescence images.

### Hematoxylin and eosin (H&E) staining

White adipose tissue (WAT), brown adipose tissue (BAT), and liver tissue were fixed in formalin and embedded in paraffin. The tissues were sectioned into 5-μm sections and stained with H&E solution. Images were captured using a pathological slide scanner (Pannoramic Scan Ⅱ, 3D Histech, Budapest, Hungary). The size of at least 100 adipocytes per mouse was quantified by employing Cellpose (https://www.cellpose.org/) and ImageJ (Version 1.8, NIH, Bethesda, MD).

### Immunofluorescence (IF) staining

After mice underwent transcardial perfusion with PBS and 4% PFA for fixation, the brains were collected and submerged in 4% PFA at 4 ℃ for 6–8 h for further fixation. The fixed brains were sequentially immersed in 20% and 30% sucrose buffers for cryoprotection at 4 ℃. Then, the brain tissue was embedded in an OCT embedding agent (Sakura, USA), and frozen sections with a thickness of 20 μm were prepared. The slices were attached to Adhesion Microscope Slides (Citotest). For the staining procedure, the slices were blocked in blocking buffer for 1 h at room temperature, followed by an overnight incubation with primary antibodies at 4 ℃. After being washed in 1× PBS, the slices were incubated in the dark with fluorescence-conjugated secondary antibodies (Cat. #A-31572 and Cat. #A-31573, Invitrogen) for 1 h at room temperature. The slices were washed again with PBS solution and then incubated with DAPI solution (1 μg/mL, Cat. #C0060, Solarbio, China) for 10 min. Subsequently, the slices were mounted using antifade mounting medium (Cat. #S2100, Solarbio). Primary antibodies included HA-Tag (C29F4) rabbit monoclonal antibody (mAb) (1∶800, Cat. #3724, Cell Signaling Technology, Boston, MA, USA) and Phospho-CREB (Ser133) Recombinant Rabbit mAb (1∶100, Cat. #BS43043, Bioworld, Nanjing, Jiangsu, China).

### Quantitative reverse transcription-PCR (RT-qPCR) analysis

Total RNA was extracted from the hypothalamus using TRIzol reagent (Cat. #9109, Takara, Dalian, Liaoning, China). The mRNA was then reverse transcribed into cDNA using a Reverse Transcription kit (Cat. #R323-01, Vazyme, Nanjing, Jiangsu, China). Quantitative PCR was performed on a real-time PCR system (Thermo Fisher Scientific, Waltham, MA, USA) using the SYBR qPCR Master Mix pre-mixed reagent (Cat. #Q331-02/03, Vazyme). The relative mRNA levels were determined using the 2^−∆Ct^ method, where ∆Ct is the difference in Ct values between the target gene and β-actin. The primers for the detected genes are listed in ***Table 1***.

**Table 1:** Primers used for quantitative reverse transcription-PCR

Genes	Sense primers (5′-3′)	Antisense primers (5′-3′)
*Raf1*	GATGCGGTGTTTGATGGCTC	CCATTCCGCACATTGACCAC
*Agrp*	ATGCTGACTGCAATGTTGCTG	CAGACTTAGACCTGGGAACTCT
*Npy*	ATGCTAGGTAACAAGCGAATGG	TGTCGCAGAGCGGAGTAGTAT
*Pomc*	ATGCCGAGATTCTGCTACAGT	TCCAGCGAGAGGTCGAGTTT

### Western blot analysis

For the Western blotting analysis of the hypothalamus, each group contained *n* ≥ 3 mouse tissues. For the Western blotting analysis of the N42 cell line, each group contained three independent cell cultures. Total protein was extracted from the tissues using tissue lysis buffer containing protease and phosphatase inhibitors (Cat. #HY-K0010, HY-K0021, HYK0022, China). Equal quantities of protein were separated by SDS-PAGE and transferred onto polyvinylidene fluoride (PVDF) membranes (Millipore, Boston, MA, USA). The membranes were blocked with 5% non-fat milk and incubated overnight at 4 ℃ with primary antibodies, including mouse anti-RAF1 mAb (1∶1000, Cat. #66592-1-Ig, Proteintech, Wuhan, China), rabbit anti-ERK1/2 polyclonal antibody (1∶500, Cat. #BS2265, Bioworld, China), rabbit anti-ERK1/2 (phospho-T202/Y204) polyclonal antibody (1∶5000, Cat. #AP0484, Bioworld), rabbit anti-MEK1/2 polyclonal antibody (1∶500, Cat. #BS3599, Bioworld), rabbit anti-MEK1/2 (phospho-S2218/222) polyclonal antibody (1∶500, Cat. #BS4733, Bioworld), rabbit anti-CREB1 polyclonal antibody (1∶1000, Cat. #12208-1-AP, Proteintech), anti-CREB (phospho S133) (1∶1000, Cat. #ab32096, Abcam, Cambridge, UK), and mouse anti-β-actin mAb (1∶5000, Cat. #BS6007M, Bioworld).

After overnight incubation in a shaker at 4 ℃, goat anti-mouse IgG (H+L) HRP (1∶10000, Cat. #BS12478, Bioworld) and goat anti-rabbit IgG (H+L) HRP (1∶10000, Cat. #BS13278, Bioworld) were incubated simultaneously. Subsequently, the PVDF membranes were treated with Enhanced Chemiluminescence Reagent (Cat. #e1070, LEBLEAD, Beijing, China) and exposed to Tanon-5260 Multi automatic chemiluminescence image analysis system (Tanon, Shanghai, China). Finally, ImageJ (Ver. 1.8, NIH, Bethesda, MD) was used for quantification in Western blotting analysis. Protein levels were normalized to those of β-actin, and phosphorylated protein levels were normalized to total protein levels.

### Statistical analysis

All data were analyzed using Prism 10.1.2 (GraphPad, San Diego, CA, USA). The statistical analyses employed in this study included Student's *t*-test, one-way ANOVA, two-way ANOVA with Bonferroni's post hoc test, or multiple *t*-tests. All data were consistent with the assumptions of the statistical tests used. All statistical tests were conducted at a significance level of *P* < 0.05. Unless otherwise stated, calculated values were expressed as mean ± standard error of the mean (SEM). Statistically significant differences are indicated by ^*^P < 0.05, ^**^P < 0.01, and ^***^P < 0.001. Statistical tests and parameter reporting are shown in the legends. Biorender (https://www.biorender.com/) was used to create schematic figures.

## Results

### The expression of *Raf1* was upregulated in hypothalamic AgRP neurons of obese mice

**Figure 1:**
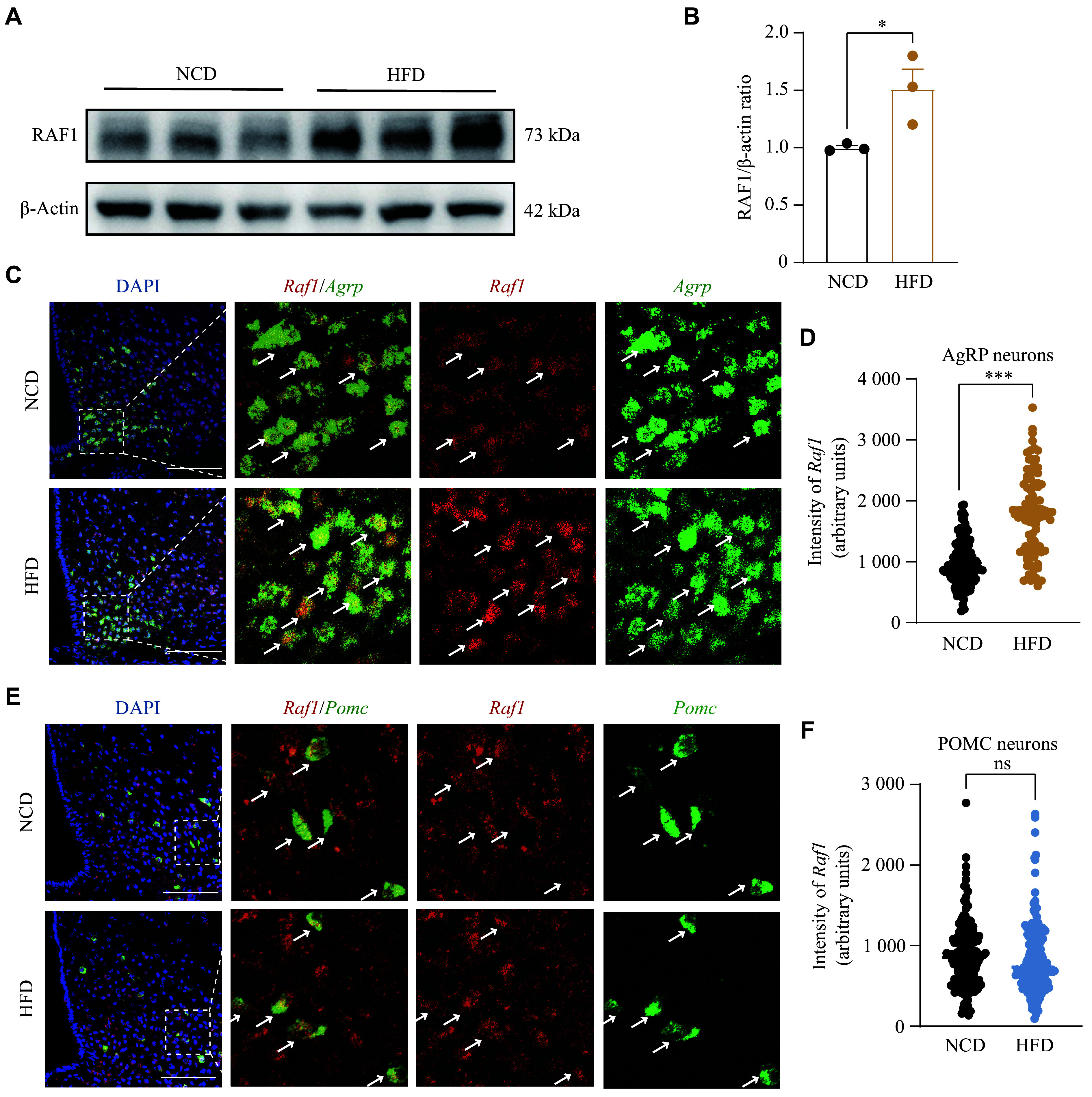
The expression of *Raf1* in AgRP neurons of DIO mice was significantly elevated. A and B: Western blotting analysis of the protein levels of RAF1 in the hypothalamus of mice fed an NCD or an HFD (*n* = 3 mice). C: Representative FISH staining shows co-localization of *Raf1* and *Agrp* mRNA in the hypothalamus of mice fed an NCD or an HFD (*n* = 3 mice; scale bars, 100 μm). D: Quantification of relative fluorescence intensity of *Raf1* mRNA FISH staining in AgRP neurons of NCD- and HFD-fed mice (*n* = 3 mice; NCD, *N* = 149; HFD, *N* = 96). E: Representative FISH staining shows co-localization of *Raf1* and *Pomc* mRNA in the hypothalamus of mice fed an NCD or an HFD (scale bars, 100 μm). Arrows in C and E indicate the co-localization of the genes. F: Quantification of relative fluorescence intensity of *Raf1* mRNA FISH staining in POMC neurons of NCD- and HFD-fed mice (*n* = 3 mice; NCD, *N* = 166; HFD, *N* = 176). *N* represents the cell number, and *n* represents the mouse number. Data are presented as the mean ± standard error of the mean. ^*^P < 0.05 and ^***^P < 0.001 by unpaired Student's *t*-tests and non-parametric tests (B, D, and F). Abbreviations: AgRP, agouti-related peptide; DIO, diet-induced obesity; FISH, fluorescence *in situ* hybridization; HFD, high-fat diet; NCD, normal chow diet; ns, not significant; RAF1, v-raf-leukemia viral oncogene 1; POMC, pro-opiomelanocortin.

To investigate whether DIO affects the expression of RAF1 in the hypothalamus, we examined the expression of RAF1 in the hypothalamus of both NCD and HFD feeding mice by Western blotting. Our results revealed that RAF1 protein levels were significantly higher in HFD mice than in NCD mice (***Fig. 1A*** and ***1B***). Next, we investigated whether the expression of *Raf1* in POMC and AgRP neurons exhibits a similar trend to that observed in the whole hypothalamus. We used FISH to examine *Raf1* mRNA in POMC or AgRP neurons in both NCD and HFD mice. Our results demonstrated that *Raf1* mRNA expression was significantly upregulated in AgRP neurons after 12 weeks of HFD feeding, compared with that in the NCD group (***Fig. 1C*** and ***1D***), while no significant change was observed in POMC neurons (***Fig. 1E*** and ***1F***). These findings suggest that HFD feeding increases *Raf1* expression specifically in AgRP neurons, but not in POMC neurons, indicating that *Raf1* in AgRP neurons may be involved in energy metabolism.

### Established a mouse model with specifically high *Raf1* expression in hypothalamic AgRP neurons

**Figure 2:**
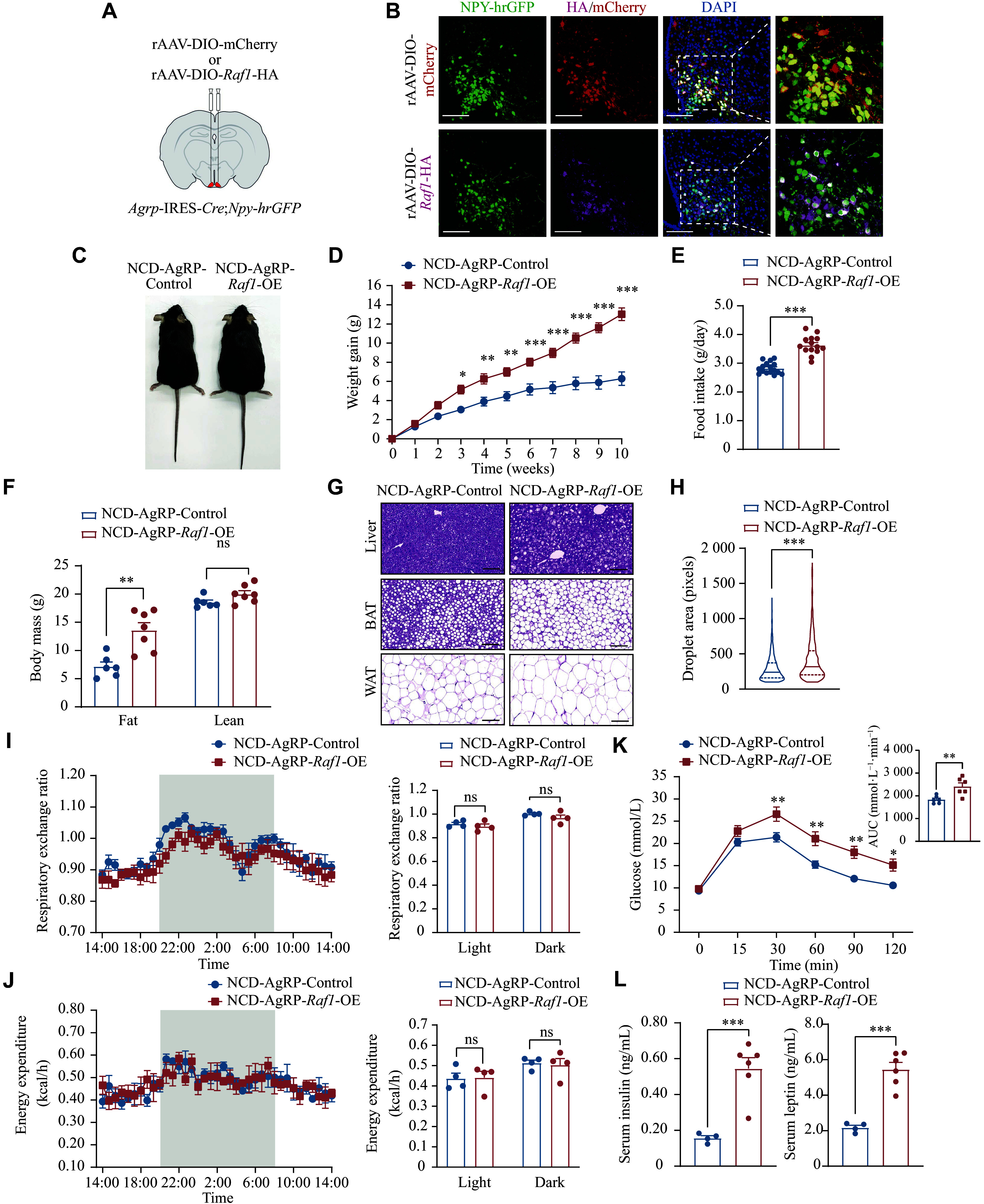
Overexpression of *Raf1* in AgRP neurons promoted obesity and related metabolic disorders. A: Schematic diagram of bilateral injections of AAV-DIO-*Raf1*-HA and its control AAV-DIO-mCherry into the ARC of *Agrp*-*IRES*-*Cre*;*Npy*-*hrGFP* mice. B: Representative IF staining of mCherry and RAF1-HA in AgRP neurons (*n* = 3 mice; scale bars, 100 μm). C: Representative image of control and AgRP-*Raf1*-OE mice fed an NCD. D–L: Various metabolic indicators, including body weight gain curves (*n* = 8–9 mice; D), food intake (*n* = 8–9 mice; E), body mass (*n* = 6–7 mice; F), representative H&E staining images of liver, BAT, and WAT (*n* = 3 mice; scale bars: 100 μm; G), droplet area of WAT (*n* = 3 mice; H), respiratory exchange ratio (*n* = 4 mice; I), energy expenditure (*n* = 4 mice; J), GTT (*n* = 6 mice; K), and serum insulin and leptin levels (*n* = 4–6 mice; L) in both control and AgRP-*Raf1*-OE mice fed an NCD were measured. Data are presented as the mean ± standard error of the mean. ^*^P < 0.05, ^**^P < 0.01, and ^***^P < 0.001 by unpaired *t*-tests (E, H, and L), two-way ANOVA with Bonferroni's post hoc test (D and K), and multiple *t*-tests (F, I, and J). Abbreviations: AgRP, agouti-related peptide; ARC, arcuate nucleus; BAT, brown adipose tissue; DIO, double-floxed inverted open reading frame; GTT, glucose tolerance test; H&E: hematoxylin and eosin; IF, immunofluorescence; NCD, normal chow diet; NPY, neuropeptide Y; ns, not significant; rAAV-DIO-*Raf1*/mCherry, recombinant adeno-associated virus encoding *Raf1*-HA or mCherry; RAF1, v-raf-leukemia viral oncogene 1; OE, overexpression; WAT, white adipose tissue.

We generated *Agrp*-*IRES*-*Cre*;*Npy*-*hrGFP* mice by breeding *Agrp*-*IRES*-*Cre* mice with *Npy*-*hrGFP* mice. In these mice, AgRP/NPY neurons were labeled with enhanced green fluorescence. Next, we injected AAVs encoding *Raf1*-HA or mCherry (AAV-DIO-*Raf1*-HA/mCherry) into the ARC of eight-week-old male *Agrp*-*IRES*-*Cre*;*Npy*-*hrGFP* mice to specifically over-express *Raf1* or mCherry in AgRP neurons, generating AgRP-*Raf1*-overexpressing (OE) mice and AgRP-mCherry control mice (***Fig. 2A***). IF staining revealed HA signal co-localized with the green fluorescence in AgRP neurons, confirming successful, high-level expression of exogenous RAF1. In contrast, control mice showed only mCherry expression in AgRP neurons (***Fig. 2B***). Thus, we established a mouse model with specifically high RAF1 expression in hypothalamic AgRP neurons.

### AgRP-*Raf1*-OE mice exhibited a typical obesity phenotype under NCD feeding

We analyzed metabolic indicators and observed that, under NCD feeding, AgRP-*Raf1*-OE mice exhibited significant weight gain, increased food intake, and notable increases in fat mass, as well as other obesity-related phenotypes (***Fig. 2C***–***2F*** and ***Supplementary Fig. 1***). Notably, *Raf1* overexpression specifically in AgRP neurons led to a marked increase in body weight, primarily due to increased fat mass rather than lean mass (***Fig. 2F***). Histological analysis of peripheral tissues revealed hepatocyte ballooning in AgRP-*Raf1*-OE mice, marked whitening of BAT, and a substantial enlargement of adipocytes in subcutaneous WAT (***Fig. 2G***). Quantitative analysis of lipid droplet size in WAT revealed that droplets were significantly larger in AgRP-*Raf1*-OE mice than in controls (***Fig. 2H***). Energy expenditure and respiratory exchange ratio remained unchanged six weeks after viral injection (***Fig. 2I*** and ***2J***). However, glucose tolerance was significantly impaired in mice with prolonged *Raf1* overexpression (***Fig. 2K***), accompanied by substantial increases in serum insulin and leptin levels (***Fig. 2L***). These findings highlight the role of *Raf1* overexpression in AgRP neurons in driving obesity-related metabolic dysfunctions under NCD feeding.

### AgRP-*Raf1* overexpression in mice modestly accelerated the development of obesity under HFD feeding

**Figure 3:**
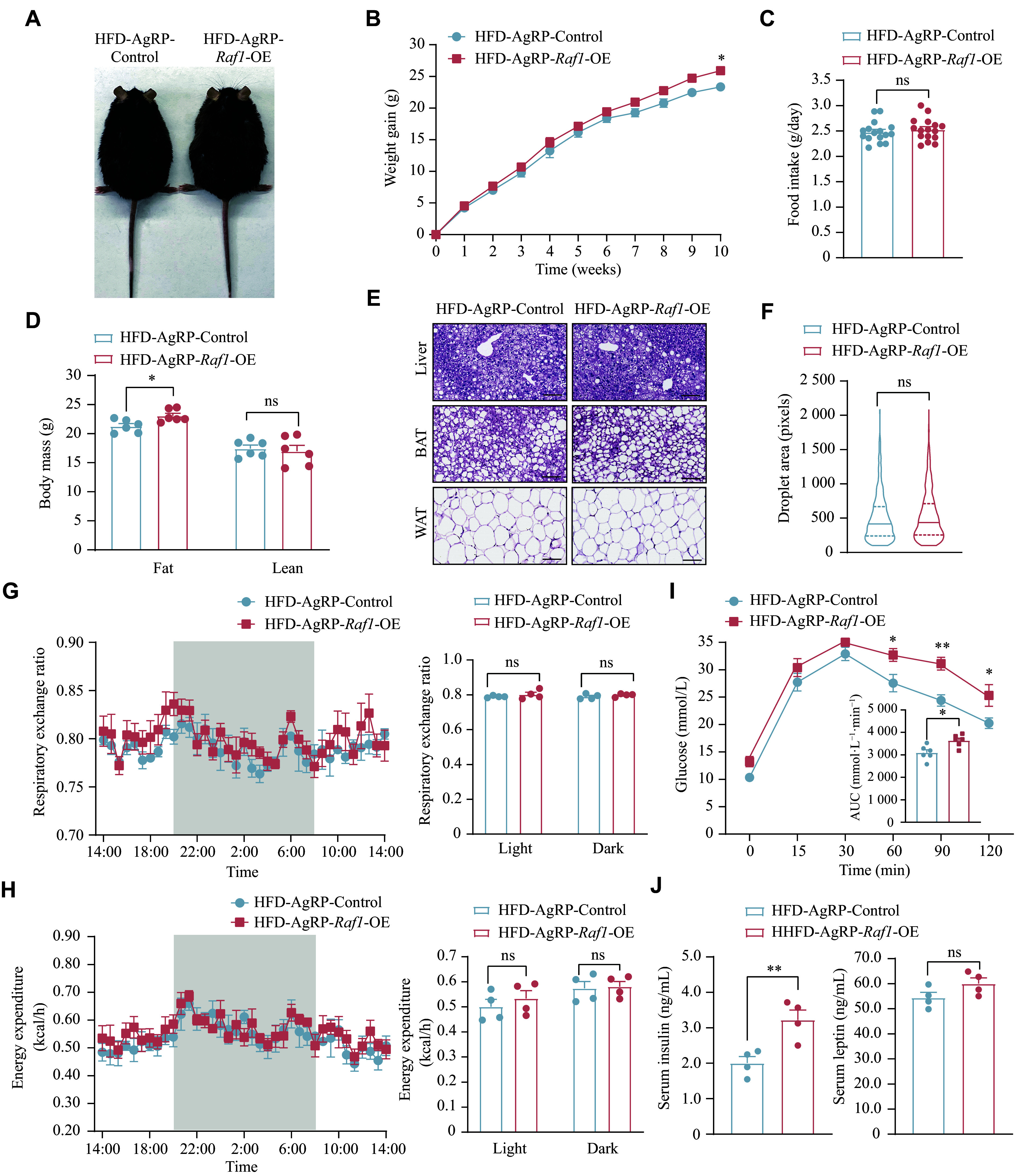
Slight promotion of obesity development was observed in AgRP-*Raf1*-OE mice under HFD feeding. A: Representative image of control and AgRP-*Raf1*-OE mice fed an HFD. B–J: Various metabolic indicators including body weight gain curves (*n* = 10 mice; B), food intake (*n* = 10 mice; C), body mass (*n* = 6 mice; D), representative H&E staining images (liver, BAT, and WAT) (*n* = 3 mice; scale bars, 100 μm; E), droplet area of WAT (*n* = 3 mice; F), respiratory exchange ratio (*n* = 4 mice; G), energy expenditure (*n* = 4 mice; H), GTT (*n* = 6 mice; I), and serum insulin and leptin levels (*n* = 4 mice; J) in both control and AgRP-*Raf1*-OE mice fed an HFD were measured. Data are presented as the mean ± standard error of the mean. ^*^P < 0.05 and ^**^P < 0.01 by unpaired Student's *t*-tests and nonparametric tests (C, F, and J), two-way ANOVA with Bonferroni's post hoc test (B and I), and multiple *t*-tests (D, G, and H). Abbreviations: AgRP, agouti-related peptide; BAT, brown adipose tissue; DIO, double-floxed inverted open reading frame; GTT, glucose tolerance test; H&E, hematoxylin and eosin; HFD, high-fat diet; ns, not significant; OE, overexpression; RAF1, v-raf-leukemia viral oncogene 1; WAT, white adipose tissue.

To further investigate the role of RAF1 in AgRP neurons in regulating energy homeostasis, AgRP-*Raf1*-OE and control mice were subjected to HFD feeding for 10 weeks (***Fig. 3A***). Interestingly, AgRP-*Raf1*-OE mice under HFD feeding displayed only mild increases in body weight gain, fat mass, and serum insulin levels compared with control mice (***Fig. 3B***, ***3D***, and ***3J***). However, AgRP-*Raf1*-OE mice showed more significant impairment of glucose tolerance compared with control mice (***Fig. 3I***). No significant differences were observed between AgRP-*Raf1*-OE mice and control mice in spontaneous food intake, histological alterations in peripheral tissues, energy expenditure, respiratory exchange ratio, or serum leptin levels (***Fig. 3C***, ***3E***–***3H***, and ***3J***). Overall, these findings indicate that elevated expression of *Raf1* in AgRP neurons in HFD-fed mice results in a mild increase in obesity and related metabolic disorders.

### Targeted knockdown of *Raf1* in AgRP neurons using CRISPR-Cas9 technology

**Figure 4:**
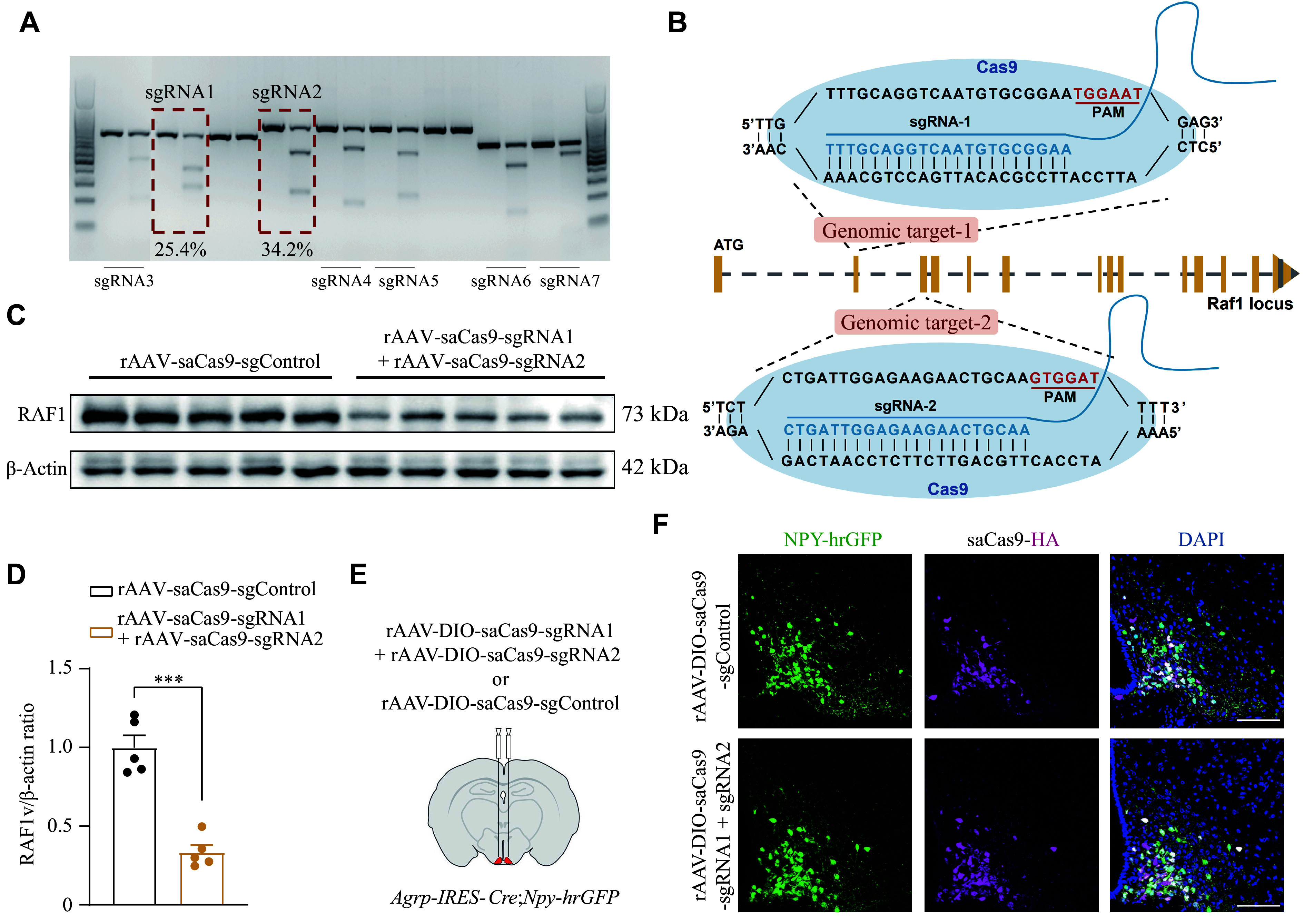
Screening and *in vivo* validation of sgRNA. A: Gel electrophoresis shows the cleavage effect of sgRNA targeting the *Raf1* gene. B: The schematic diagrams show sgRNA1 and sgRNA2. C and D: Western blotting analysis of RAF1 protein levels in the hypothalamus after control and knockdown AAV injection. β-Actin served as the loading control (*n* = 5 mice). rAAV-saCas9-sgRNA1, rAAV-saCas9-sgRNA2, and rAAV-saCas9-sgControl (recombinant adeno-associated viruses encoding sgRNA1, sgRNA2, and a non-targeting control sgRNA, respectively) were used. E: A schematic diagram of bilateral injection of Cre-dependent AAV-DIO-saCas9-sgRNAs and its control into the ARC of *Agrp*-*IRES*-*Cre*;*Npy*-*hrGFP* mice. F: Representative IF staining of the HA-Tag signifying the expression of saCas9 in AgRP neurons (*n* = 3 mice; scale bars, 100 μm). Data are presented as the mean ± standard error of the mean. ^***^P < 0.001 by unpaired *t*-tests (D). Abbreviations: AgRP, agouti-related peptide; ARC, arcuate nucleus; DIO, double-floxed inverted open reading frame; NPY, neuropeptide Y; RAF1, v-raf-leukemia viral oncogene 1; sgRNA, single guide RNA.

Next, we investigated whether specific knockdown of *Raf1* in AgRP neurons results in a metabolic phenotype opposite to that observed in AgRP-*Raf1*-OE mice. We designed seven sgRNAs specifically targeting exons of the mouse *Raf1* gene. These sgRNAs were individually cloned into the lentiviral transfer vector pCK002_U6-Sa-sgRNA(mod)_EFS-SaCas9-2A-Puro_WPRE^[25–27]^, from which we generated lentiviral particles and subsequently infected N42 cells. Notably, the N42 cells used here served only to elucidate RAF1 function in this specific pathway, representing a general hypothalamic model rather than AgRP neurons. One week post-infection, we performed T7EI assays to analyze the mutation frequencies of N42 cells infected with lentiviruses carrying sgRNAs. The results showed that lenti-sgRNA1 and lenti-sgRNA2 exhibited the highest mutation rates in N42 cells (***Fig. 4A***).

To determine the *in vivo* gene knockdown efficiency of sgRNA1 and sgRNA2, we cloned them into an AAV vector: pX601-AAV-CMV::NLS-SaCas9-NLS-3xHA-bGHpA;U6::BsaI-sgRNA and generated AAV-saCas9-sgRNA1 and AAV-saCas9-sgRNA2 viruses^[28–30]^ (***Fig. 4B***). Then, the AAVs carrying sgRNA1, sgRNA2, or control guide RNA (sgControl) were stereotaxically injected into the hypothalamus of 8-week-old male C57BL/6J mice. Deletion of the *Raf1* gene was validated by Western blotting three weeks after virus injection (***Fig. 4C***), showing that RAF1 expression in the hypothalamus of mice infected with AAV-saCas9-sgRNA1 or AAV-saCas9-sgRNA2 decreased by approximately 70% compared with controls (***Fig. 4D***), thereby confirming efficient *in vivo* gene editing.

To induce AgRP-specific knockout of *Raf1*, we cloned sgRNA1 and sgRNA2 into an AAV vector comprising double-floxed inverted open reading frame and saCas9 (pAAV-FLEXDIO-SaCas9-U6-sgRNA)^[31]^ and produced the corresponding AAV-DIO-saCas9-sgRNA1 and AAV-DIO-saCas9-sgRNA2 viruses. Then, eight-week-old male *Agrp*-*IRES*-*Cre*;*Npy*-*hrGFP* mice underwent bilateral stereotaxic injections of AAVs into the ARC (***Fig. 4E***). The AAVs expressing sgRNA targeting *Raf1* harbored Cre-dependent HA-Tag, whereas the control AAVs expressed only Cre-dependent saCas9^[32–33]^. The expression of AAV-DIO-saCas9 viruses was verified by IF staining, demonstrating co-localization of the HA-Tag with hrGFP in AgRP/NPY neurons. The exclusive expression of saCas9-HA-Tag in AgRP neurons suggested the successful generation of AgRP-specific *Raf1* knockout (AgRP-*Raf1*-KO) mice (***Fig. 4F***).

### AgRP-*Raf1*-KO mice maintained normal body weight on NCD feeding but were protected against DIO on HFD feeding

**Figure 5:**
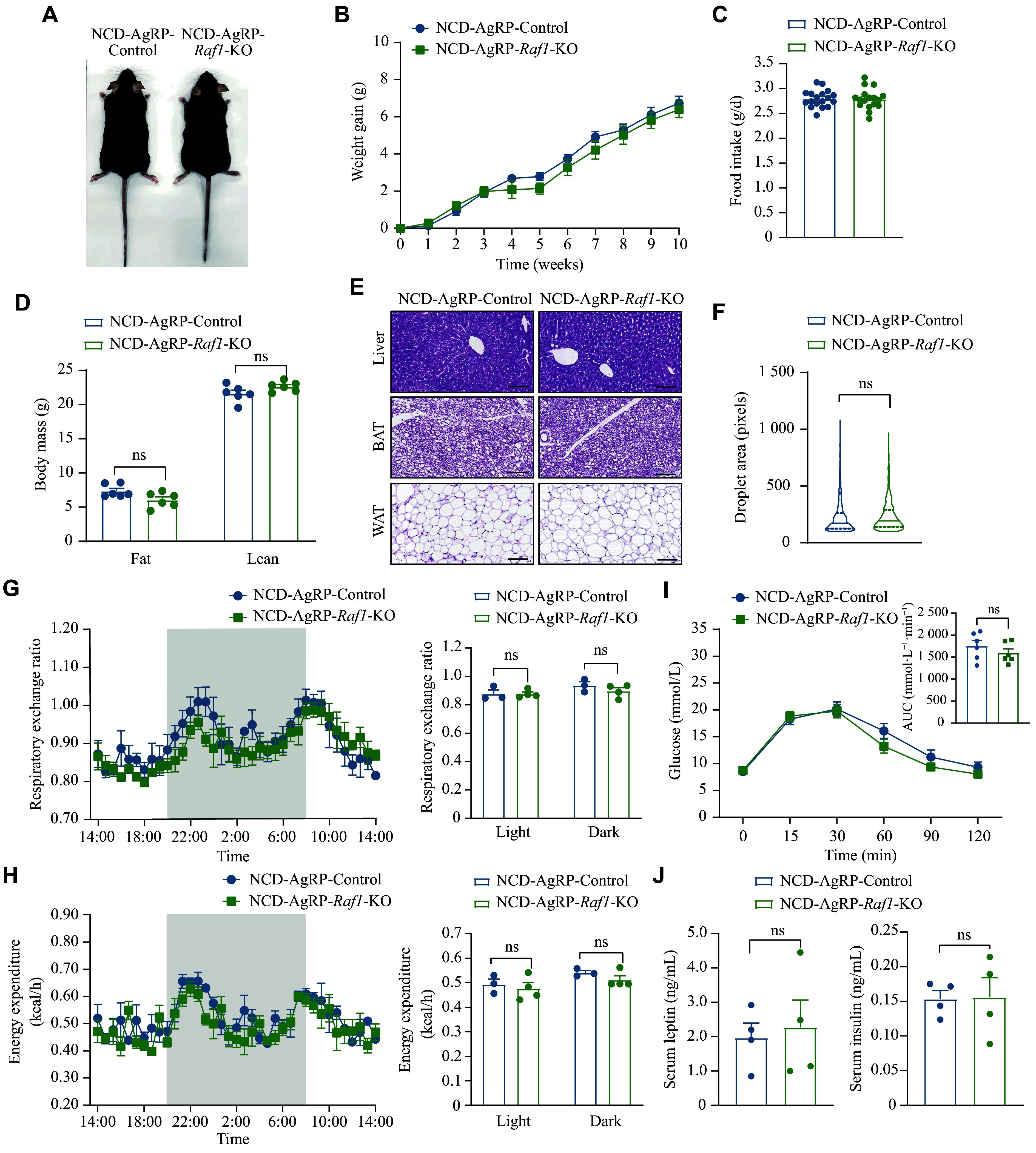
*Raf1* knockout in AgRP neurons in mice did not alter their metabolic phenotypes under NCD feeding. A: Representative image of control and AgRP-*Raf1*-KO mice fed an NCD. B–J: Various metabolic indicators including body weight gain curves (*n* = 8 mice; B), food intake (*n* = 8 mice; C), body mass (*n* = 6 mice; D), representative H&E staining images (liver, BAT, and WAT) (*n* = 3 mice; scale bars, 100 μm; E), droplet area of WAT (*n* = 3 mice; F), respiratory exchange ratio (*n* = 3–4 mice; G), energy expenditure (*n* = 3–4; H), GTT (*n* = 6 mice; I), and serum insulin and leptin levels (*n* = 4 mice; J) in both control and AgRP-*Raf1*-KO mice fed an NCD were measured. Data are presented as the mean ± standard error of the mean. ^ns^*P* > 0.05 by unpaired Student's *t*-tests and nonparametric tests (C, F, and J), two-way ANOVA with Bonferroni's post hoc test (B and I), and multiple *t*-tests (D, G, and H). Abbreviations: AgRP, agouti-related peptide; ARC, arcuate nucleus; BAT, brown adipose tissue; DIO, diet-induced obesity; GTT, glucose tolerance test; H&E, hematoxylin and eosin; KO, knockout; NCD, normal chow diet; ns, not significant; RAF1, v-raf-leukemia viral oncogene 1; WAT, white adipose tissue.

After establishing the AgRP-*Raf1*-KO mouse model, we examined the metabolic phenotypes of AgRP-*Raf1*-KO mice under both NCD and HFD feeding. Contrary to our expectations, the AgRP-*Raf1*-KO mice did not exhibit any significant changes in body weight gain compared with the control mice when fed an NCD (***Fig. 5A*** and ***5B***). Similarly, other metabolic phenotypes, including spontaneous food intake, fat mass, energy expenditure, respiratory exchange ratio, glucose tolerance, serum insulin level, and serum leptin level, showed no significant difference between AgRP-*Raf1*-KO and control mice when fed an NCD (***Fig. 5C***–***5J***).

**Figure 6:**
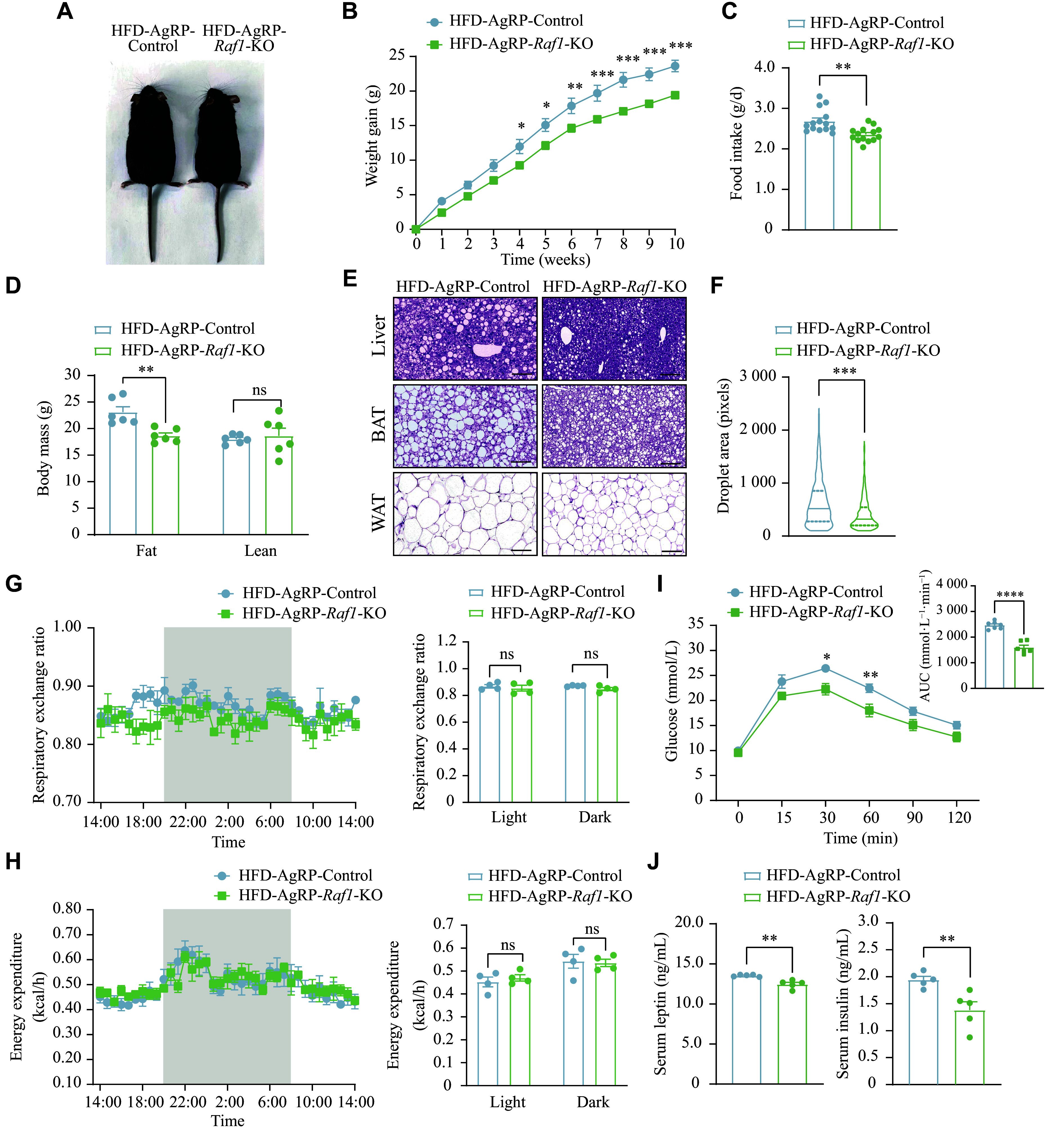
*Raf1* knockout in AgRP neurons protected against DIO. A: Representative image of control and AgRP-*Raf1*-KO mice fed an HFD. B–J: Various metabolic indicators, including body weight gain curves (*n* = 8–10 mice; B), food intake (*n* = 10 mice; C), body mass (*n* = 6 mice; D), representative H&E staining images (liver, BAT, and WAT) (*n* = 3 mice; scale bars, 100 μm; E), droplet area of WAT (*n* = 3 mice; F), respiratory exchange ratio (*n* = 4 mice; G), energy expenditure (*n* = 4 mice; H), GTT (*n* = 6–7 mice; I), and serum insulin and leptin levels (*n* = 4 mice; J) in both control and AgRP-*Raf1*-KO mice fed an HFD were measured. Data are presented as the mean ± standard error of the mean. ^*^P < 0.05, ^**^P < 0.01, and ^***^P < 0.01 by unpaired Student's *t*-tests and nonparametric tests (C, F, and J), two-way ANOVA with Bonferroni's post hoc test (B and I), and multiple t-tests (D, G, and H). Abbreviations: AgRP, agouti-related peptide; ARC, arcuate nucleus; BAT, brown adipose tissue; DIO, diet-induced obesity; GTT, glucose tolerance test; H&E, hematoxylin and eosin; HFD, high-fat diet; KO, knockout; ns, not significant; RAF1, v-raf-leukemia viral oncogene 1; WAT, white adipose tissue.

Notably, when fed an HFD, the AgRP-*Raf1*-KO mice exhibited significantly lower body weight gain and food intake compared with control mice (***Fig. 6A***–***6C***). The lower body weight gain was attributed to a smaller increase in fat mass rather than lean mass increase in AgRP-*Raf1*-KO mice (***Fig. 6D***). Furthermore, hepatocytic ballooning was alleviated in AgRP-*Raf1*-KO mice. Concurrently, the whiteness of BAT was reduced, and the size of adipose cells in subcutaneous WAT was also significantly decreased in AgRP-*Raf1*-KO mice (***Fig. 6E*** and ***6F***). There were no significant differences in energy expenditure and respiratory exchange ratio between AgRP-*Raf1*-KO and control mice (***Fig. 6G*** and ***6H***). Glucose tolerance was significantly improved in long-term AgRP-*Raf1*-KO mice, compared with the control mice (***Fig. 6I***). The deficiency of *Raf1* signaling in AgRP neurons impacted serum hormone homeostasis in HFD-fed mice, with both insulin and leptin levels showing a pronounced decrease (***Fig. 6J***). Collectively, our data demonstrate that specific *Raf1* knockout in AgRP neurons confers protection against DIO.

### The RAF1-MEK1/2-ERK1/2-CREB axis of the MAPK pathway regulated the expression of *Agrp* and *Npy*

**Figure 7:**
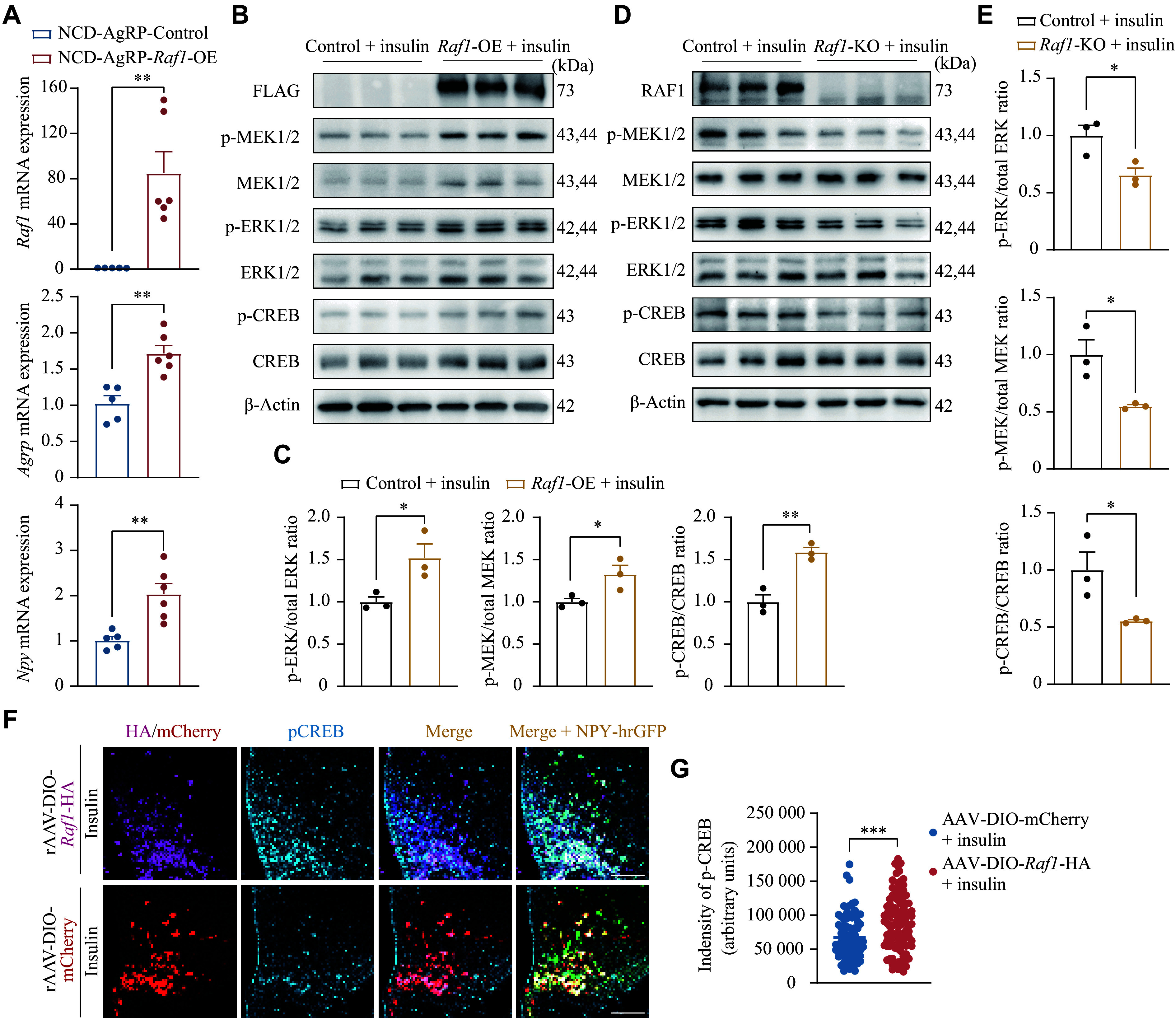
Raf1 regulated MAPK signaling under insulin stimulation. A: Relative mRNA levels of *Raf1*, *Agrp*, and *Npy* in the hypothalamus of control and AgRP-*Raf1*-OE mice fed an NCD (*n* = 5–6 mice). B: Western blotting analysis of protein levels of FLAG, MEK1/2, pMEK1/2, ERK1/2, pERK1/2, CREB, and pCREB in the N42 *Raf1*-overexpression cells. β-Actin served as the internal control (*n* = 3). C: Phosphorylation levels of MEK1/2, ERK1/2, and CREB proteins in the N42 *Raf1*-overexpression cells. D: Western blotting analysis of protein levels of RAF1, MEK1/2, pMEK1/2, ERK1/2, pERK1/2, and pCREB in the N42 *Raf1*-knockout cells. β-Actin served as the internal control (*n* = 3). E: Phosphorylation levels of MEK1/2, ERK1/2, and CREB proteins in the N42 *Raf1*-knockout cells. F: Representative IF staining of pCREB in AgRP neurons of control and AgRP-*Raf1*-OE mice following 2 mU insulin (icv) stimulation (*n* = 3 mice; scale bars, 100 μm). G: Fluorescence intensity quantification of pCREB co-localized with HA/mCherry (*n* = 3 mice; AAV-DIO-mCherry, *N* = 77; AAV-DIO-*Raf1*-HA, *N* = 105). *N* represents the cell number, and *n* represents the mouse number. Data are presented as the mean ± standard error of the mean. ^*^P < 0.05, ^**^P < 0.01, and ^***^P < 0.001 by unpaired *t*-tests (A, C, E, and G). Abbreviations: AgRP, agouti-related peptide; CREB, cAMP response element-binding protein; ERK1/2, extracellular signal-regulated kinases 1 and 2; icv, intra-cerebroventricular injection; IF, immunofluorescence; MEK1/2, mitogen-activated protein kinase kinases 1 and 2; NCD, normal chow diet; NPY, neuropeptide Y; OE, overexpression; p-CREB, phospho-CREB; p-ERK1/2, phospho-ERK1/2; p-MEK1/2, phospho-MEK1/2; RAF1, v-raf-leukemia viral oncogene 1.

To investigate how *Raf1* regulates energy metabolism in AgRP neurons, we used RT-qPCR to analyze the expression of *Agrp* and *Npy* in the hypothalamus of AgRP-*Raf1*-OE mice. Results showed significantly elevated expression of both genes in the hypothalamus of AgRP-*Raf1*-OE mice compared with control mice (***Fig. 7A***), suggesting that RAF1 promotes the expression of *Agrp* and *Npy*. As a classical serine/threonine protein kinase, RAF1 plays a critical role in the MAPK signaling pathway^[34–35]^. To investigate whether RAF1 regulates the expression of *Agrp* and *Npy* *via* the MAPK signaling pathway, we established both *Raf1*-overexpressing and *Raf1*-knockout N42 cell lines. In these cell lines, we investigated the phosphorylation levels of mitogen-activated protein kinase kinases 1 and 2 (MEK1/2) and extracellular signal-regulated kinases 1 and 2 (ERK1/2) upon insulin stimulation^[36–38]^.

Western blotting analysis revealed that phosphorylation levels of MEK1/2 and ERK1/2 were significantly increased in *Raf1*-overexpressing N42 cells compared with control cells (***Fig. 7B*** and ***7C***). Conversely, in *Raf1*-knockout N42 cells, insulin stimulation led to a marked reduction in the phosphorylation levels of MEK1/2 and ERK1/2 compared with control cells (***Fig. 7D*** and ***7E***).

Studies have shown that cAMP response element-binding protein (CREB), a downstream signal of *Raf1* and ERK1/2, activated *via* phosphorylation at Ser133^[39–41]^, regulates the transcription of *Agrp* and *Npy* in AgRP neurons^[42–43]^. Thus, we also detected the phosphorylation level of CREB in both *Raf1*-overexpressing N42 cells and *Raf1*-knockout N42 cells upon insulin stimulation. Similar to MEK1/2 and ERK1/2, insulin stimulation significantly increased the phosphorylation level of CREB in *Raf1*-overexpressing N42 cells, while it was markedly reduced in N42 cells with *Raf1* knockout (***Fig. 7B***–***7E***).

**Figure 8:**
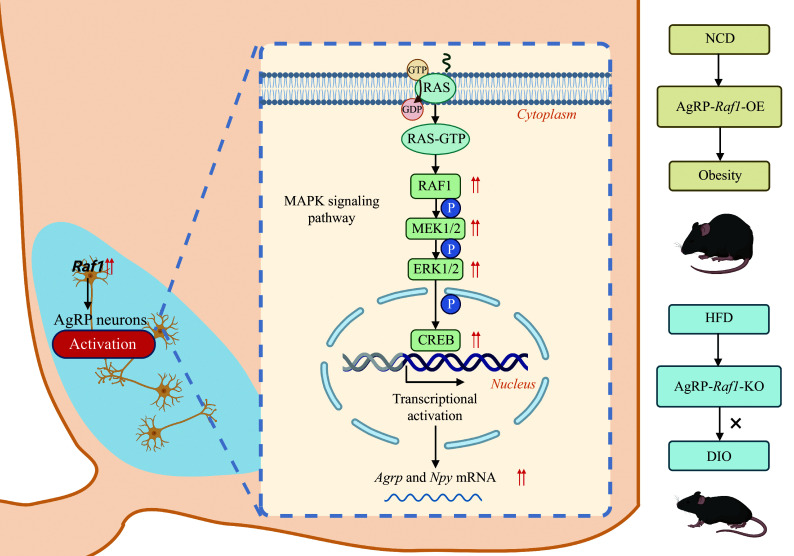
Role of hypothalamic AgRP neuron *Raf1* in energy homeostasis regulation. This graphic abstract illustrates the pivotal role of *Raf1* within hypothalamic AgRP neurons in governing energy homeostasis. Specifically, *Raf1* exerts its regulatory function *via* the MAPK-mediated modulation of *Agrp* and *Npy* expression. In normal physiological conditions, this mechanism contributes to the maintenance of energy balance. Under HFD challenges, the RAF1-MAPK-AGRP/NPY axis becomes dysregulated, leading to disruptions in energy homeostasis. Abbreviations: DIO, diet-induced obesity; HFD, high-fat diet; KO, knockout; OE, overexpression.

In line with the *in vitro* findings, we observed significant increases in CREB phosphorylation in the AgRP neurons of insulin-stimulated AgRP-*Raf1*-OE mice (***Fig. 7F*** and ***7G***), confirming that insulin effectively activates the RAF1-MEK1/2-ERK1/2-CREB axis of the MAPK signaling pathway in these neurons. Taken together, our results suggest that *Raf1* in AgRP neurons regulates energy metabolism by modulating the expression of *Agrp* and *Npy* through the MAPK signaling pathway^[44–45]^. This aligns with our qPCR data showing elevated *Agrp* and *Npy* expression in the hypothalamus of AgRP-*Raf1*-OE mice, indicating that the increased CREB phosphorylation likely underlies this upregulation. Consequently, *Raf1* within AgRP neurons appears to regulate food intake and obesity by enhancing the expression of *Agrp* and *Npy* through CREB phosphorylation (***Fig. 8***).

## Discussion

The global obesity prevalence poses a major health challenge, contributing to chronic diseases such as diabetes and cardiovascular conditions^[46]^. Obesity results from disruptions in energy balance, a process regulated by the hypothalamus, which integrates hormonal and nutritional signals to control food intake and energy expenditure^[47–48]^. In the ARC, POMC neurons suppress appetite and increase energy expenditure^[9,49]^, while AgRP neurons stimulate appetite and reduce energy expenditure^[50–51]^. The balance between these neurons is vital for metabolic health, but disruptions can lead to obesity^[12,52]^. Further research is warranted to elucidate the underlying molecular mechanisms, particularly the key genes implicated in the regulation of energy metabolism. *Raf1*, widely recognized as an oncogene, has been extensively studied for its critical role in tumorigenesis and cellular proliferation over the years^[53–54]^. Acting as a serine/threonine kinase and a central component of the MAPK signaling pathway, *Raf1* is a well-established mediator of cell growth, survival, and differentiation^[55–56]^. While its role in cancer biology remains a predominant focus, emerging evidence highlights its potential involvement in other physiological processes, particularly energy metabolism. For example, pharmacological inhibition of ERK1/2, a key downstream target of RAF1, has been shown to improve insulin sensitivity in mice^[57–58]^. However, despite these findings, the specific metabolic function of RAF1 in key metabolism-related neurons of the hypothalamus has remained unexplored until now.

In the present study, we aimed to address this knowledge gap by examining the role of RAF1 in hypothalamic neurons involved in metabolic regulation under both an NCD and an HFD. Notably, we observed a significant increase in RAF1 levels in the AgRP neurons of mice with DIO. This observation underscores the potential role of RAF1 in AgRP neurons in regulating energy metabolism. To validate our hypothesis, we employed recombinant AAVs to induce targeted overexpression or knockout of *Raf1* specifically in AgRP neurons.

The overexpression of *Raf1* in AgRP neurons resulted in a pronounced obese phenotype, even under NCD conditions. Mice with AgRP-*Raf1* overexpression exhibited significant weight gain, mild hyperphagia, increased adiposity, and impaired glucose tolerance. These findings indicate that *Raf1* overactivation in AgRP neurons disrupts normal energy and glucose homeostasis, potentially through the dysregulation of downstream signaling pathways. Under HFD, these mice exhibited only mild increases in body weight and adiposity. This may be attributed to the fact that the HFD overshadows the inherent function of *Raf1*. The HFD regimen concurrently leads to an elevation in body weight, and the effect of *Raf1* is less remarkable than that of the HFD. Meanwhile, the HFD may saturate the downstream MAPK-CREB signaling pathway or desensitize the activity of AgRP neurons, thus rendering the metabolic phenotype less pronounced than that under NCD feeding. Conversely, *Raf1* knockout in AgRP neurons conferred resistance to DIO under HFD conditions. These *Raf1*-deficient mice displayed reduced weight gain, decreased food intake, lower fat mass, and improved glucose tolerance compared with their wild-type counterparts. The stark phenotypic differences between the overexpression and knockout models firmly establish *Raf1* as a critical regulator of energy balance and glucose metabolism within AgRP neurons.

At the mechanistic level, our findings reveal that an HFD significantly elevates *Raf1* expression in the hypothalamus, which in turn activates the RAF1-MEK1/2-ERK1/2-CREB axis of the MAPK signaling pathway in AgRP neurons. This pathway plays a pivotal role in modulating the expression of neuropeptides such as AGRP and NPY, which are integral to appetite regulation and energy expenditure. The hyperactivation of this signaling cascade disrupts peripheral glucose and lipid homeostasis, contributing to the development of obesity and related metabolic disorders. By contrast, disrupting *Raf1* signaling in AgRP neurons appears to mitigate these effects, highlighting the therapeutic potential of targeting *Raf1* in metabolic interventions.

The implications of these findings are twofold. First, they underscore the previously unrecognized role of *Raf1* in hypothalamic energy regulation, specifically in AgRP neurons. This adds a new dimension to our understanding of *Raf1*'s physiological functions beyond its well-characterized role in cancer biology. Second, they identify RAF1 as a potential therapeutic target for combating obesity and metabolic disorders. Given the growing prevalence of obesity and its associated health complications, including type 2 diabetes and cardiovascular diseases, there is an urgent need for novel therapeutic strategies. Targeting the RAF1-MEK1/2-ERK1/2-CREB axis of the MAPK signaling pathway in the hypothalamus may provide a promising avenue for the development of interventions aimed at restoring metabolic balance.

Our study focuses on the role of the *Raf1* gene in regulating metabolism within AgRP neurons, and recent findings on p53 function in the same neuronal population provide valuable context for comparison. It has been reported that p53 ablation in AgRP neurons leads to increased hypothalamic JNK activity and subsequent DIO^[59]^. Thus, the role of p53, a well-known tumor suppressor, in AgRP neurons provides a reference for our study. In future research, the combination of our research findings with p53-related studies reveals the complexity of AgRP neuron signaling and highlights the importance of dissecting the molecular network that regulates the metabolic response to dietary challenges.

Our study demonstrates that *Raf1* in AgRP neurons regulates energy metabolism *via* the MAPK pathway. However, a key limitation must be acknowledged: the metabolic phenotype following *Raf1* deletion, resistance to DIO and improved glucose tolerance, is not unique to RAF1 disruption. This mirrors findings from prior research targeting other genes in AgRP neurons (*e.g.*, *Foxo1*, *Atf4*, *Ghsr*), suggesting a broader pattern: impairing intracellular signaling in AgRP neurons generally attenuates their orexigenic activity, thereby protecting against metabolic dysfunction. While our work identifies *Raf1* as a novel component in this network, the non-unique phenotype highlights that AgRP neurons may exhibit limited redundancy in maintaining pro-obesity functions. Future studies should explore whether *Raf1* exerts pathway-specific effects distinct from other signaling molecules in AgRP neurons to clarify its unique role. Due to the more stable metabolic phenotypes of male mice compared with female mice^[60]^, male mice are preferable for metabolic research. However, the absence of female cohorts may limit the generalizability of our findings, an issue that will be addressed in future studies.

In conclusion, the present study provides compelling evidence that *Raf1* in hypothalamic AgRP neurons plays a pivotal role in regulating energy homeostasis. By modulating the expression of *Agrp* and *Npy* through the MAPK signaling pathway, *Raf1* contributes to the maintenance of energy balance under normal conditions and mediates metabolic dysfunction in response to an HFD. These findings not only enhance our understanding of the molecular mechanisms underlying obesity but also highlight RAF1 as a promising target for therapeutic strategies aimed at mitigating metabolic diseases. Future research should focus on further elucidating the upstream regulators of *Raf1* in AgRP neurons and exploring potential pharmacological interventions to modulate this pathway in a cell-specific manner.

## Additional information

The online version contains supplementary materials available at http://www.jbr-pub.org.cn/article/doi/10.7555/JBR.39.20250114.
