# Supplementary material for: RAF1 in AgRP neurons involved in the regulation of energy metabolism via the MAPK signaling pathway
Source: J Biomed Res. 2025 May 28;40(1):45–62. doi: 10.7555/JBR.39.20250114 (PMC12794213; doi:10.7555/JBR.39.20250114)
Supplement: Supplementary file 1 — The online version contains supplementary materials available at http://www.jbr-pub.org.cn/article/doi/10.7555/JBR.39.20250114. [file jbr-40-1-45-S1.pdf]

Available online at www.jbr-pub.org.cn

Open Access at PubMed Central

JBR

Journal of Biomedical Research, 2026 40(1): 1–1

Supplementary data

RAF1 in AgRP neurons involved in the regulation of energy metabolism *via* the MAPK signaling pathway

Yuqian Chen[1,2,△], Lianci Ren[1,3,△], Xinyi Xu[1,2,△], Zhenning Sun[1,2], Mingxi Dai[1], Yin Li[1,2], Xiang Ma[1,3,✉], Juxue Li[1,2,4,5,✉]

[1]*State Key Laboratory of Reproductive Medicine and Offspring Health, Nanjing Medical University, Nanjing, Jiangsu 211166, China;*
[2]*Jiangsu Provincial Key Laboratory of Molecular Targets and Intervention of Metabolic Disease Nanjing Medical University, Nanjing, Jiangsu 211166, China;*
[3]*Clinical Center for Reproductive Medicine, the First Affiliated Hospital of Nanjing Medical University, Nanjing, Jiangsu 210029, China;*
[4]*The Affiliated Eye Hospital, Nanjing Medical University, Nanjing, Jiangsu 210029, China;*
[5]*The Second Affiliated Hospital of Nanjing Medical University, Nanjing, Jiangsu 210011, China.*

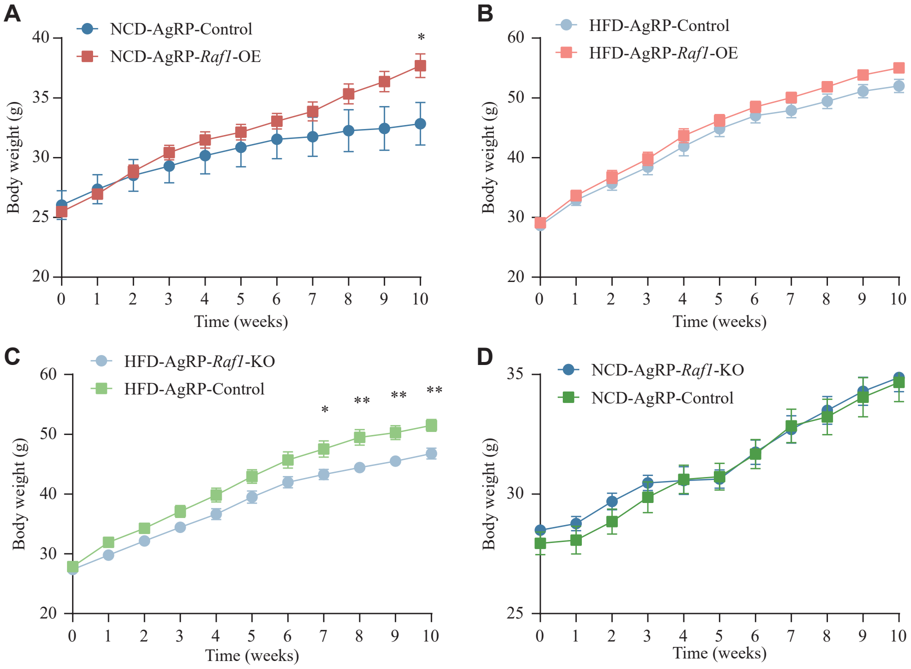

Supplementary Fig. 1 **Body weight of mice with genetic modification of *Raf1* in AgRP neurons.** A and B: Body weight of control and AgRP-*Raf1*-OE mice fed an NCD (A; n = 10 mice) or an HFD (B; n = 10 mice). C and D: Body weight of control and AgRP-*Raf1*-KO mice fed an HFD (C; n = 10 mice) or an NCD (D; n = 8 mice). Data are presented as the mean ± standard error of the mean. $^{*}P$ < 0.05 and $^{**}P$ < 0.01 by two-way ANOVA with Bonferroni's post hoc test.

△These authors contributed equally to this work.

✉Corresponding authors: Juxue Li and Xiang Ma, State Key Laboratory of Reproductive Medicine and Offspring Health, 101 Longmian Avenue, Nanjing Medical University, Nanjing, Jiangsu 211166, China; E-mail: lijuxue@njmu.edu.cn (Li) and sxmaxiang@126.com (Ma).

Received: 18 March 2025; Revised: 15 May 2025; Accepted: 20 May 2025; Published online: 28 May 2025

CLC number: R589.2, Document code: A

The authors reported no conflict of interests.

This is an open access article under the Creative Commons Attribution (CC BY 4.0) license, which permits others to distribute, remix, adapt and build upon this work, for commercial use, provided the original work is properly cited.

© 2026 by Journal of Biomedical Research.

https://doi.org/10.7555/JBR.39.20250114
